# Pistachio Leaf Extract Modulates Redox-Dependent Mitochondrial and Metabolic Responses in Ethanol-Stressed HepG2 Cells

**DOI:** 10.3390/ijms27114836

**Published:** 2026-05-27

**Authors:** Enrico La Spina, Emanuela Tropea, Annalisa Santisi, Alfio Distefano, Lucia Longhitano, Giuseppe Antonio Malfa, Cesarina Giallongo, Giuseppe Alberto Palumbo, Giovanni Li Volti, Daniele Tibullo, Ignazio Alberto Barbagallo

**Affiliations:** 1 Department of Biotechnological and Biomedical Sciences, University of Catania, 95123 Catania, Italy; enricolaspina@outlook.it (E.L.S.); tropeaemanuela3@gmail.com (E.T.); distalfio@gmail.com (A.D.); lucia.longhitano@unict.it (L.L.); cesarina.giallongo@unict.it (C.G.); livolti@unict.it (G.L.V.); d.tibullo@unict.it (D.T.); ignazio.barbagallo@unict.it (I.A.B.); 2 Department of Medical and Surgical Sciences and Advanced Technologies “G.F. Ingrassia”, University of Catania, 95123 Catania, Italy; giuseppe.palumbo@unict.it; 3 Department of Drug Science—Biochemistry Section, University of Catania, Viale A. Doria 6, 95125 Catania, Italy; giuseppe.malfa@unict.it

**Keywords:** *Pistacia vera* L., redox homeostasis, alcohol metabolism, hepatocellular homeostasis, EtOH-induced cytotoxicity

## Abstract

Ethanol-induced hepatocellular injury is characterized by redox imbalance and mitochondrial dysfunction, which critically determine cellular adaptation to metabolic stress. Rather than acting as simple antioxidants, effective nutraceutical strategies may enhance endogenous adaptive responses that preserve cellular homeostasis. Pistachio (*Pistacia vera* L.) leaves, an underexplored agricultural by-product, represent a potential source of bioactive compounds with redox-modulating properties. In this study, we investigated the effects of pistachio leaf extract (PLE) in human HepG2 hepatocytes exposed to 1% ethanol (EtOH). EtOH reduced cell viability and increased intracellular and mitochondrial reactive oxygen species (ROS), while triggering compensatory responses including glutathione accumulation and activation of metabolic and inflammatory pathways. PLE alone induced a mild cytosolic ROS signal and increased PGC-1α expression, consistent with transcriptional changes potentially associated with hormetic activation of mitochondrial regulatory pathways. Notably, co-treatment with EtOH and PLE significantly reduced both cytosolic and mitochondrial ROS levels, prevented excessive glutathione accumulation and was associated with transcriptional changes consistent with improved glutathione-related adaptive responses. These effects were associated with transcriptional modulation of genes involved in mitochondrial biogenesis and metabolic adaptation, including PGC-1α, TFAM, SIRT1, PPARα, and UCP2. In parallel, PLE attenuated EtOH-induced IL-6 expression without preventing FAS upregulation, indicating a selective dissociation between inflammatory and lipogenic responses. Overall, our findings suggest that PLE may act as a modulator of redox-dependent signaling rather than as a direct antioxidant and is associated with transcriptional and functional signatures consistent with mitochondrial adaptive responses to ethanol-induced stress. These results support the potential of pistachio leaf-derived phytocomplexes as nutraceutical modulators of hepatocellular adaptive responses.

## 1. Introduction

Alcohol consumption represents a major global health burden and a leading cause of chronic liver disease, largely driven by disruption of hepatocellular redox homeostasis and mitochondrial function [1,2,3]. At the cellular level, EtOH metabolism profoundly alters hepatocellular homeostasis by increasing ROS production, perturbing GSH-dependent redox balance, impairing mitochondrial function, and activating inflammatory pathways [4,5,6]. Because hepatocytes rely heavily on mitochondrial oxidative metabolism and redox control [7], mitochondrial adaptability represents a critical determinant of cellular resilience to EtOH-induced stress [8]. In recent years, increasing attention has been devoted to nutritional and nutraceutical strategies aimed at reinforcing endogenous adaptive responses rather than merely scavenging ROS [9]. In this context, metabolic flexibility has been previously demonstrated for leaf-derived phytocomplexes modulating cellular metabolic responses [10,11,12,13,14,15]. Importantly, many phytochemicals exert their beneficial effects through hormetic mechanisms, characterized by mild redox stimulation that primes antioxidant defenses and improves cellular stress tolerance [16,17,18]. Pistacia vera L. is widely appreciated for the nutritional and health-promoting properties of its kernels [19]. However, pistachio cultivation also generates large amounts of agricultural by-products that remain largely underutilized [20]. Pistachio trees are deciduous, and their leaves naturally fall during autumn and winter before re-emerging in late spring [21]. As a consequence, pistachio leaves are considered a seasonal agricultural waste, despite being rich in polyphenols, flavonoids, and phenolic acids with potential antioxidant and anti-inflammatory activity [22,23]. The pistachio cultivated in the Bronte area (Sicily, Italy) is internationally renowned for its unique nutritional characteristics, which are closely linked to the volcanic soils of Mount Etna [24]. While the kernels of Bronte pistachio have been extensively studied and valorized [25], its leaves represent an untapped bioresource.

The recovery and functional exploitation of pistachio leaves align with circular bioeconomy principles and sustainable valorisation of agricultural by-products, offering the opportunity to transform a low-value seasonal waste into a source of bioactive compounds with potential nutraceutical relevance [26,27,28,29,30].

Although the antioxidant properties of pistachio leaf extracts have been described in chemical and food matrices [26,31,32], their effects at the cellular level, particularly in the context of EtOH-induced hepatic stress, remain poorly understood. It is unclear whether pistachio leaf-derived bioactives act as simple antioxidants or whether they modulate adaptive pathways involving redox regulation, mitochondrial remodeling, and inflammatory signaling. We have recently reported a detailed chemical characterization of PLE, demonstrating a complex phytochemical profile dominated by polyphenols with recognized redox activity [33]. While the chemical composition of PLE has been established [34,35,36], its biological effects may vary depending on the cellular context and experimental model. In the present study, the same chemically characterized extract was investigated in human HepG2 hepatocytes focusing on EtOH-induced oxidative stress, mitochondrial adaptation, and inflammatory responses, with the goal of elucidating the molecular mechanisms underlying its potential hepatoprotective and nutraceutical value.

## 2. Results

### 2.1. Effect of PLE and EtOH on HepG2 Cell Viability

**Figure 1:**
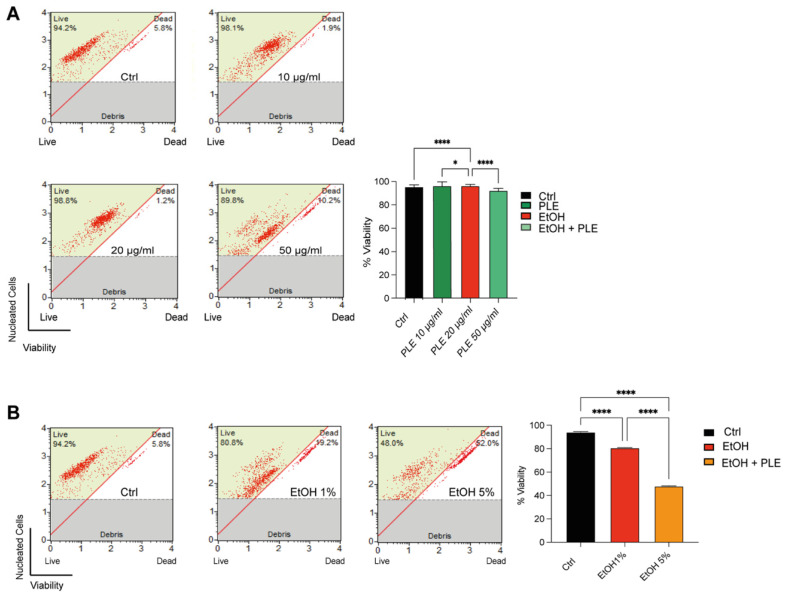
Cytotoxicity profiling of PLE and EtOH in HepG2 cells. (**A**) Representative Muse™ Cell Analyzer dot plots and quantitative analysis of HepG2 cell viability following 24 h treatment with increasing concentrations of PLE (10, 20, and 50 μg/mL). (**B**) Representative dot plots and quantification of cell viability after 24 h exposure to increasing EtOH concentrations (1% and 5%). Data are presented as mean ± SD of independent experiments as described in the Methods section. Statistical analysis was performed using one-way ANOVA followed by Tukey’s multiple comparison post hoc test. Statistical significance was defined as * *p* < 0.05, and **** *p* < 0.0001.

To evaluate PLE cytotoxic potential, HepG2 cells were treated for 24 h with increasing concentrations of PLE (10, 20, and 50 μg/mL). As shown in Figure 1A, none of the tested PLE concentrations significantly affected cell viability compared with untreated cells, indicating that PLE is non-cytotoxic within this concentration range. The effect of EtOH on HepG2 cell viability was then assessed by exposing cells to increasing concentrations for 24 h. As shown in Figure 1B, EtOH induced a significant reduction in cell viability in a dose-dependent manner. Importantly, 1% EtOH caused a clear and reproducible decrease in viable cells while maintaining overall cellular integrity. Based on this dose–response analysis, 1% EtOH was selected for all subsequent experiments, as it induced a significant but sub-cytotoxic reduction in cell viability, allowing the investigation of EtOH-induced oxidative stress and mitochondrial dysfunction without extensive cell death.

### 2.2. PLE Attenuates EtOH-Induced Loss of Cell Viability

**Figure 2:**
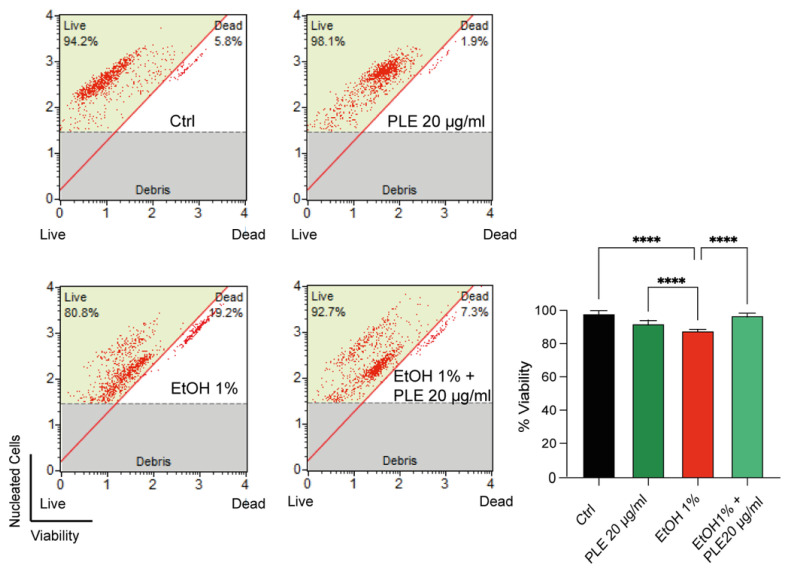
PLE attenuates EtOH-induced cytotoxicity in HepG2 cells. Representative Muse™ Cell Analyzer dot plots and quantitative analysis of cell viability following 24 h treatment with EtOH (1%), PLE (20 μg/mL), or their combination. Data are presented as mean ± SD of independent experiments as described in the Methods section. Statistical analysis was performed using one-way ANOVA followed by Tukey’s multiple comparison post hoc test. Statistical significance was defined as **** *p* < 0.0001.

To investigate whether PLE may counteract EtOH-induced cytotoxicity, HepG2 cells were exposed to 1% EtOH in combination or not with PLE (20 μg/mL). As shown in Figure 2, treatment with 1% EtOH significantly reduced cell viability compared with control cells, confirming the deleterious effects of EtOH on hepatocyte survival. Co-treatment with PLE markedly attenuated the EtOH-induced reduction in cell viability. In particular, HepG2 cells treated with EtOH in the presence of PLE displayed a significant increase in the percentage of viable cells compared with EtOH-treated cells alone, restoring viability values close to those observed in control conditions. Treatment with PLE alone did not significantly affect cell viability, confirming the absence of cytotoxic effects of the extract under the experimental conditions used. Having established the safety profile of PLE in HepG2 cells, we next investigated whether the extract could modulate cellular redox homeostasis, particularly under ethanol-induced stress.

### 2.3. PLE Reshapes Glutathione Dynamics and Redox-Related Gene Expression in EtOH-Exposed Cells

**Figure 3:**
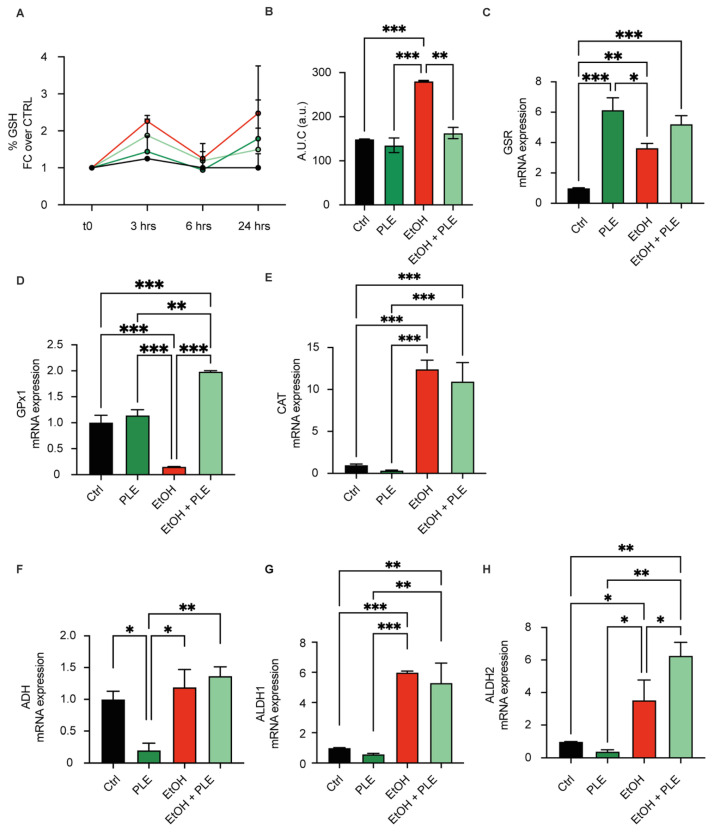
PLE reshapes glutathione dynamics and glutathione-dependent antioxidant responses in EtOH-exposed HepG2 cells. (**A**) Time-course analysis of intracellular reduced glutathione (GSH) levels in HepG2 cells treated with EtOH (1%), PLE (20 μg/mL), or their combination over a 24 h period. Data are expressed as fold change over control (t0). (**B**) Area under the curve (AUC) analysis of GSH levels over 24 h, integrating cumulative glutathione changes during treatments. (**C**–**H**) Relative mRNA expression levels of GSR, GPX1, CAT, ADH, ALDH1, and ALDH2 following 24 h treatments, assessed by quantitative real-time PCR and normalized to the housekeeping gene GAPDH. Data are presented as mean ± SD of independent experiments as described in the Methods section. Statistical analysis was performed using one-way ANOVA followed by Tukey’s multiple comparison post hoc test. Statistical significance was defined as * *p* < 0.05, ** *p* < 0.01, and *** *p* < 0.001.

Intracellular reduced GSH levels were measured in HepG2 cells treated with EtOH, in combination or not with PLE (Figure 3A,B). As shown in Figure 3A, EtOH treatment induced a significant increase at 3 and 24 h in intracellular GSH levels compared with control cells. PLE treatment alone did not significantly affect GSH levels. Co-treatment with EtOH and PLE resulted in significantly lower GSH levels compared with EtOH-treated cells across the time course. Consistently, area under the curve (AUC) analysis (Figure 3B) showed that cumulative GSH levels were significantly increased by EtOH and significantly reduced by co-treatment with PLE. The expression of GSH-related antioxidant enzymes was subsequently analyzed. Glutathione reductase (GSR) mRNA expression was significantly increased by PLE treatment alone and remained elevated in EtOH + PLE-treated cells (Figure 3C). EtOH treatment alone induced a modest increase in GSR expression. In contrast, EtOH significantly reduced glutathione peroxidase 1 (GPx1) expression (Figure 3D), while co-treatment with PLE restored GPx1 expression to levels significantly higher than those observed in EtOH-treated cells. Catalase (CAT) expression was significantly increased by EtOH treatment and was not significantly modified by PLE co-treatment (Figure 3E).

### 2.4. PLE Modulates Ethanol-Metabolizing Pathways in Hepatocytes

Given that oxidative stress in ethanol-exposed hepatocytes is closely linked to ethanol metabolism and acetaldehyde generation, we next evaluated the expression of enzymes involved in ethanol detoxification (Figure 3F–H). Alcohol dehydrogenase (ADH) expression was not significantly altered by EtOH exposure compared with control cells, whereas PLE treatment alone significantly reduced ADH levels. Under co-treatment conditions, ADH expression increased relative to PLE-treated cells, restoring values close to those observed in control cells. Aldehyde dehydrogenase 1 (ALDH1) expression was significantly induced by EtOH exposure and remained unchanged upon co-treatment with PLE, indicating that the extract did not further modulate this isoform. In contrast, aldehyde dehydrogenase 2 (ALDH2) expression was significantly increased by EtOH treatment and was further upregulated in the presence of PLE, suggesting a transcriptional adaptive response potentially relevant to mitochondrial acetaldehyde metabolism under co-treatment conditions.

### 2.5. PLE Modulates ROS Homeostasis and Is Associated with Mitochondrial Adaptive Responses in EtOH-Exposed Cells

**Figure 4:**
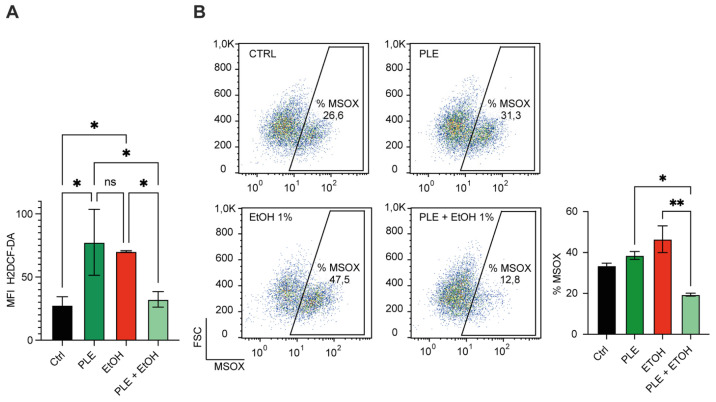
Effects of PLE on intracellular and mitochondrial ROS in EtOH-exposed HepG2 cells. (**A**) Quantification of total intracellular ROS levels expressed as mean fluorescence intensity (MFI) relative to control conditions following 24 h treatments. (**B**) Quantification of mitochondrial superoxide production expressed as percentage of MitoSOX-positive cells following 24 h treatments. Data are presented as mean ± SD of independent experiments as described in the Methods section. Statistical analysis was performed using one-way ANOVA followed by Tukey’s multiple comparison post hoc test. Statistical significance was defined as *ns* = *not significant*, * *p* < 0.05, and ** *p* < 0.01.

**Figure 5:**
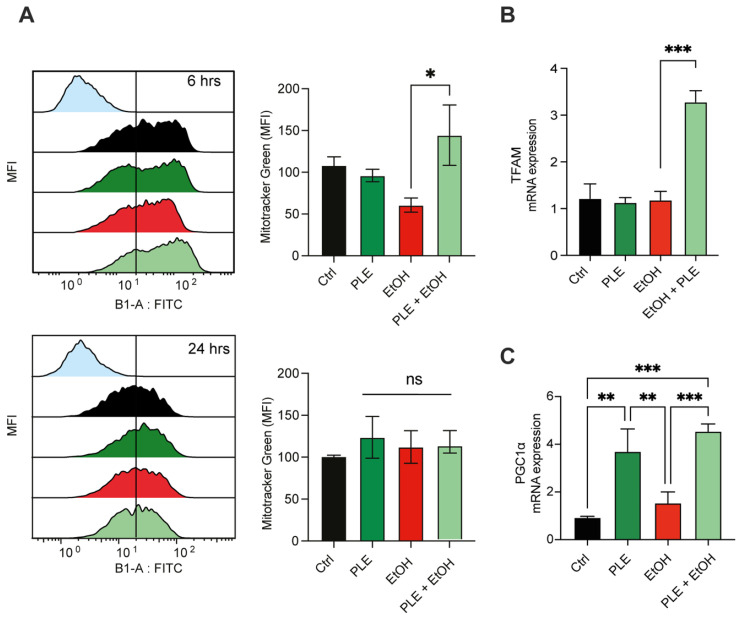
Effects of PLE on mitochondrial mass and biogenesis-related gene expression in EtOH-exposed HepG2 cells. (**A**) Representative flow cytometry histograms and quantitative analysis of mitochondrial mass assessed by MitoTracker™ Green FM staining at 6 h and 24 h. Mitochondrial mass is expressed as mean fluorescence intensity (MFI) relative to control conditions. (**B**,**C**) Relative mRNA expression levels of TFAM and PGC-1α following 24 h treatments. Data are presented as mean ± SD of independent experiments as described in the Methods section. Statistical analysis was performed using one-way ANOVA followed by Tukey’s multiple comparison post hoc test. Statistical significance was defined as *ns* = *not significant*, * *p* < 0.05, ** *p* < 0.01, and *** *p* < 0.001.

To evaluate the effects of PLE on ethanol-induced oxidative stress and mitochondrial adaptation, intracellular ROS levels and mitochondrial superoxide production were analysed. EtOH exposure significantly increased total intracellular ROS levels compared with control cells (Figure 4A). PLE treatment alone also induced a moderate increase in intracellular ROS relative to control conditions. In contrast, co-treatment with EtOH and PLE significantly reduced total ROS levels compared with EtOH-treated cells, restoring values toward control levels (Figure 4A). A similar trend was observed at the mitochondrial level (Figure 4B). EtOH exposure significantly increased mitochondrial superoxide production compared with control cells, while PLE treatment alone induced a modest increase. Notably, co-treatment with EtOH and PLE markedly reduced mitochondrial ROS levels compared with EtOH alone, indicating a protective effect of PLE against ethanol-induced mitochondrial oxidative stress. Given the central role of mitochondrial function in cellular redox homeostasis, we next evaluated whether PLE influenced mitochondrial biogenesis. Mitochondrial mass was not significantly altered by EtOH or PLE treatment alone compared with control cells. No substantial differences were observed at earlier time points, whereas a significant increase emerged under co-treatment conditions at the later experimental stage. However, co-treatment with EtOH and PLE significantly increased mitochondrial mass relative to EtOH-treated cells at the later time point (Figure 5A). Consistently, mitochondrial transcription factor A (TFAM) expression was not significantly modified by EtOH or PLE alone, whereas co-treatment significantly increased TFAM levels compared to all other experimental conditions (Figure 5B). In parallel, PLE treatment alone significantly increased the expression of the mitochondrial biogenesis regulator PGC-1α (Figure 5C), while EtOH alone did not significantly affect its expression. Co-treatment with EtOH and PLE also resulted in a significant increase in PGC-1α levels compared with EtOH-treated cells.

### 2.6. PLE Promotes Mitochondrial and Metabolic Adaptive Responses in EtOH-Exposed Hepatocytes

**Figure 6:**
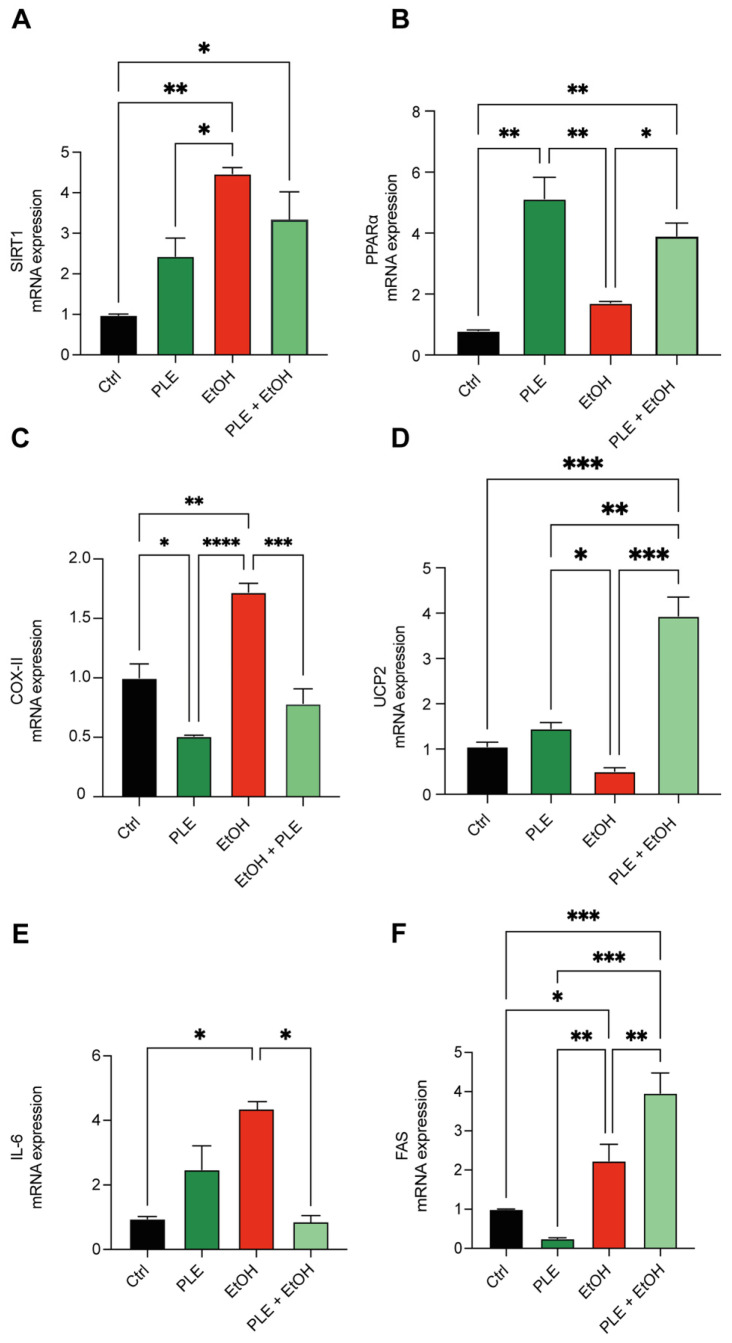
PLE differentially regulates mitochondrial, metabolic, inflammatory, and lipogenic gene expression in EtOH-exposed HepG2 cells. Relative mRNA expression of genes involved in mitochondrial regulation and metabolic adaptation is shown in panels (**A**–**D**): (**A**) SIRT1, (**B**) PPARα, (**C**) COX-II, and (**D**) UCP2. Genes associated with inflammatory and lipogenic responses are shown in panels (**E**,**F**): (**E**) IL-6 and (**F**) FAS. Data are presented as mean ± SD of independent experiments as described in the Methods section. Statistical analysis was performed using one-way ANOVA followed by Tukey’s multiple comparison post hoc test. Statistical significance was defined as * *p* < 0.05, ** *p* < 0.01, *** *p* < 0.001, and **** *p* < 0.0001.

The expression of genes involved in mitochondrial function, metabolic adaptation, and inflammatory signaling was evaluated to determine how PLE modulates hepatocellular responses to ethanol exposure. As shown in Figure 6A, EtOH treatment significantly increased sirtuin 1 (SIRT1) mRNA expression compared with control cells. PLE treatment alone also increased SIRT1 expression, although to a lesser extent than EtOH. Co-treatment with EtOH and PLE maintained SIRT1 levels above control conditions, suggesting sustained activation of adaptive metabolic signaling. Analysis of peroxisome proliferator-activated receptor alpha (PPARα) expression revealed a significant increase following PLE treatment alone compared with control cells (Figure 6B). EtOH exposure induced a more moderate increase, whereas co-treatment resulted in intermediate expression levels relative to the single treatments. Mitochondrial uncoupling protein 2 (UCP2) expression displayed a distinct pattern (Figure 6D). EtOH treatment reduced UCP2 expression compared with control cells, whereas PLE treatment alone induced a modest increase. Notably, co-treatment with EtOH and PLE resulted in a marked increase in UCP2 expression compared with all other conditions. Cytochrome c oxidase subunit II (COX-II) expression was significantly increased following EtOH treatment (Figure 6C). In contrast, PLE treatment alone reduced COX-II expression relative to control cells. Co-treatment attenuated the EtOH-induced increase, bringing expression levels closer to those observed in control conditions. In addition to mitochondrial and metabolic genes, markers of lipogenesis and inflammation were also modulated. EtOH exposure significantly increased fatty acid synthase (FAS) expression compared with control cells (Figure 6F). PLE treatment alone reduced FAS expression, whereas co-treatment with EtOH resulted in a further increase in FAS levels relative to EtOH alone. Finally, EtOH exposure markedly increased interleukin-6 (IL-6) expression (Figure 6E), while co-treatment with PLE significantly reduced IL-6 levels compared with EtOH-treated cells.

## 3. Discussion

Ethanol-induced hepatic stress arises from the coordinated disruption of redox homeostasis [37,38], mitochondrial function [39], and inflammatory signalling [40], processes regulated by adaptive cytoprotective redox pathways [41,42]. In this study, we demonstrate that PLE exerts a context-dependent modulatory effect on these processes, acting not as a conventional antioxidant but as a regulator of redox-dependent signaling.

Importantly, our findings support the emerging concept that effective nutraceutical interventions should not aim at indiscriminate suppression of reactive oxygen species, but rather at the fine-tuning of redox signaling pathways that govern mitochondrial adaptation and cellular stress responses. In line with this framework, PLE induced a mild increase in cytosolic ROS under basal conditions, consistent with a hormetic response that primes mitochondrial regulatory mechanisms without causing oxidative damage.

Under ethanol-induced stress, this priming translated into a coordinated adaptive response characterized by reduced ROS accumulation, glutathione-related adaptive responses, and enhanced activation of mitochondrial biogenesis and metabolic pathways. These findings suggest that PLE may enhance cellular resilience through redox-related and transcriptional changes consistent with mitochondrial adaptive responses rather than through direct ROS scavenging. Treatment with PLE alone induced a modest increase in cytosolic ROS without altering basal glutathione levels, a pattern indicative of controlled redox signalling rather than oxidative damage. This behaviour aligns with the concept of hormetic redox modulation, whereby mild oxidative cues activate stress-response pathways and prime antioxidant systems to operate more efficiently under subsequent challenges [43]. When administered in combination with EtOH, PLE significantly reduced intracellular ROS accumulation while preventing excessive glutathione overaccumulation. The lower glutathione levels observed under co-treatment are unlikely to reflect depletion, but rather more efficient glutathione-related adaptive responses. This interpretation is supported by the coordinated upregulation of GSR and restoration of GPx1 expression, indicating improved efficiency of GSH recycling and peroxide detoxification. The biochemical basis of these effects can be linked to the complex polyphenolic profile of PLE, which is dominated by gallic acid, quercetin and myricetin glycosides, together with minor amounts of anacardic acid derivatives (Supplementary Table S1). These compounds are increasingly recognised as modulators of redox-sensitive signalling pathways involved in mitochondrial function and metabolic adaptation [44,45,46,47,48]. Compared with previous studies mainly focused on the antioxidant properties of pistachio-derived matrices or isolated polyphenols, the present work provides cellular evidence that pistachio leaf extract may modulate redox-sensitive adaptive responses in ethanol-stressed hepatocytes. This extends previous observations by linking the phytochemical profile of PLE with coordinated changes in ROS handling, glutathione-related responses, and transcriptional markers of mitochondrial regulation. Gallic acid and flavonol derivatives such as quercetin and myricetin have been shown to induce mild and transient redox signals capable of activating mitochondrial biogenesis regulators [44,49], including SIRT1 and PGC-1α [50]. In line with this concept, PLE treatment alone increased PGC-1α expression without affecting mitochondrial mass or TFAM levels, indicating a mitochondrial PPARα priming effect under basal conditions. This priming does not translate into constitutive mitochondrial expansion, underscoring that PLE does not indiscriminately activate mitochondrial biogenesis. In contrast, under ethanol-induced stress, PLE promoted a coordinated upregulation of PGC-1α and TFAM, accompanied by a significant increase in mitochondrial mass [51]. Notably, ethanol alone did not significantly alter mitochondrial mass or biogenesis markers, indicating that PLE selectively enhances adaptability in response to stress. Although functional bioenergetic measurements were not assessed in the present study, the coordinated regulation of mitochondrial biogenesis markers is consistent with an adaptive mitochondrial response. EtOH metabolism plays a central role in shaping hepatocellular redox balance and mitochondrial integrity [52]. Over 90% of ingested EtOH is predominantly metabolized in the liver by a rate-limiting enzyme, ADH 1, to acetaldehyde, which is further metabolized to acetate by mitochondrial ALDH 2 [53]. The selective enhancement of mitochondrial ALDH2 expression observed under EtOH exposure in the presence of PLE is particularly relevant, given the pivotal role of ALDH2 in limiting acetaldehyde-induced mitochondrial toxicity in acute kidney injury [54]. At the functional level, EtOH exposure increased SIRT1 expression, reflecting an intrinsic adaptive response to metabolic stress. PLE further modulated this response under EtOH exposure, supporting transcriptional changes consistent with mitochondrial and metabolic adaptation. Similarly, PPARα and UCP2 were selectively upregulated only in the combined treatment, suggesting improved metabolic flexibility and mitigation of mitochondrial oxidative pressure. In contrast, cytochrome c oxidase subunit II expression was increased by ethanol, reduced by PLE alone, and significantly attenuated under co-treatment. This pattern indicates that PLE fine-tunes ethanol-induced respiratory chain remodelling rather than suppressing mitochondrial respiration, preventing excessive activation while preserving adaptive capacity. A notable dissociation emerged between lipogenic and inflammatory pathways. Unexpectedly, PLE did not counteract ethanol-induced FAS upregulation and, under co-treatment conditions, further increased FAS transcription. This observation indicates that the adaptive redox and mitochondrial effects associated with PLE are not necessarily paralleled by suppression of lipogenic signaling. Rather than reflecting a uniformly protective phenotype, these findings may suggest a more complex metabolic remodeling response induced by combined ethanol and phytochemical exposure. Therefore, the impact of PLE on lipid metabolic pathways requires further mechanistic investigation. While PLE effectively attenuated ethanol-induced IL-6 expression, it did not counteract FAS upregulation. This finding suggests that lipogenic signaling may be regulated independently of redox and inflammatory adaptation. Such pathway-specific modulation highlights that mitochondrial protection and inflammatory control can be uncoupled from lipid metabolic responses [55]. Beyond its biological relevance, this study also supports the valorisation of *P. vera* L., leaves as a sustainable source of bioactive compounds [32], similar to other nutritionally derived plant extracts regulating metabolic resilience [10]. The recovery and functional exploitation of pistachio leaf-derived extracts align with circular bioeconomy principles, contributing to waste reduction and the development of value-added nutraceutical ingredients. In this context, the demonstration that a leaf-derived extract can modulate hepatocellular redox balance, mitochondrial adaptability, and inflammatory signaling under ethanol-induced stress provides a strong rationale for its sustainable reuse in functional food and nutraceutical applications [29,30,31]. Overall, these findings are consistent with a hormetic mechanism, whereby mild redox stimulation primes adaptive pathways and enhances cellular resilience. These findings support a role for PLE in modulating mitochondrial and metabolic adaptation pathways beyond classical antioxidant mechanisms. This study has some limitations. First, the use of HepG2 cells, although widely adopted, may not fully recapitulate the metabolic complexity of primary hepatocytes. Second, functional assessments of mitochondrial respiration and bioenergetics were not performed, and future studies will be necessary to confirm the impact of PLE on mitochondrial function. In addition, the present study was designed as an exploratory investigation aimed at characterizing the biological effects of PLE under ethanol-induced stress conditions rather than dissecting specific signaling pathways through pharmacological intervention. Future studies including pathway-specific inhibitors and positive pharmacological controls will be necessary to further clarify the molecular mechanisms involved. Although multiple redox-related parameters were evaluated in the present study, including intracellular ROS, mitochondrial superoxide production, glutathione dynamics, and antioxidant-related gene expression, additional biochemical markers of oxidative stress and antioxidant defense (e.g., lipid peroxidation, SOD, CAT, and GPx activity) would further strengthen the mechanistic interpretation of PLE-mediated redox modulation. Furthermore, inflammatory signaling was evaluated through IL-6 expression only. Additional inflammatory mediators, including TNF-α, IL-1β, NF-κB, and COX-2, should be investigated in future studies to better define the anti-inflammatory potential of PLE under ethanol-induced stress conditions. Third, the experimental model reflects acute ethanol exposure and does not capture chronic adaptive responses occurring in vivo.

## 4. Materials and Methods

### 4.1. Reagents and Pistachio Leaf Extract

All reagents were of analytical grade. EtOH was purchased from Sigma-Aldrich (St. Louis, MO, USA). Cell culture media and supplements were obtained from Gibco (Thermo Fisher Scientific, Waltham, MA, USA). PLE was prepared from *Pistacia vera* L. leaves as previously described [33]. Briefly, dried leaves were finely ground and extracted using a water-based solvent system under controlled conditions. The extract was filtered, concentrated under reduced pressure, and lyophilized. The chemical composition of PLE was previously characterized by HPLC-DAD and UHPLC–ESI–MS in our earlier study [33]. The same extraction protocol, plant material origin, and extract batch were used in the present work. Briefly, the extract was shown to be rich in polyphenolic compounds, including flavonols, flavones, and phenolic acids. For completeness, a summary of the main identified compounds and their quantitative content is reported in Supplementary Table S1. The full analytical dataset has been published previously and is not reproduced here.

### 4.2. Cell Culture

Human hepatocellular carcinoma HepG2 cells were obtained from the American Type Culture Collection (ATCC, HB-8065, Manassas, VA, USA). Cells were cultured in Dulbecco’s Modified Eagle’s Medium (DMEM) supplemented with 10% (*v*/*v*) fetal bovine serum (FBS), 2 mM L-glutamine, 100 U mL^−1^ penicillin, and 100 μg/mL streptomycin. HepG2 cells were maintained according to standard cell culture procedures routinely used for ATCC HB-8065 cells. Cells were maintained at 37 °C in a humidified atmosphere containing 5% CO_2_ and routinely passaged at 70–80% confluence. For treatments, PLE was freshly dissolved in sterile culture medium, filtered through a 0.22 μm membrane, and added to cells. The final solvent concentration did not exceed 0.1% (*v*/*v*) and had no effect on cell viability.

### 4.3. Experimental Design and Treatments

For all experiments, HepG2 cells were seeded in 6-well or 12-well plates depending on the assay, at densities ranging between 1 × 10^5^ and 3 × 10^5^ cells/well, and allowed to adhere overnight before treatment. Culture medium volumes were adjusted according to plate format to ensure consistent experimental conditions. To evaluate PLE cytotoxicity, cells were treated for 24 h with increasing concentrations of PLE (10, 20, and 50 μg/mL). Based on dose–response viability assays, 20 μg/mL PLE was selected for subsequent experiments. To induce cellular stress, cells were exposed to increasing EtOH concentrations for 24 h. Based on viability data, 1% (*v*/*v*) EtOH was selected as a sub-cytotoxic concentration for all subsequent analyses. For combined treatments, cells were co-treated with EtOH (1%) and PLE (20 μg/mL) for 24 h. Control cells received vehicle alone.

### 4.4. Cell Viability Analysis

Cell viability was assessed using the Muse™ Cell Analyzer (Luminex Corporation, Austin, TX, USA) and the Muse™ Count & Viability Kit (Cat. No. MCH100102) according to the manufacturer’s instructions. After treatment, cells were harvested, stained, and analyzed by flow cytometry. Data are expressed as percentage of viable cells relative to control conditions.

### 4.5. Flow Cytometry Analyses

Intracellular reduced glutathione (GSH), cytoplasmic reactive oxygen species (ROS), mitochondrial superoxide production, and mitochondrial mass were analyzed by flow cytometry using a MACSQuant^®^ 14 cytometer (Miltenyi Biotec S.r.l., Bologna, BO, Italy). For GSH determination, cells were stained with ThiolTracker™ Violet (Thermo Fisher Scientific), a thiol-reactive fluorescent probe, according to the manufacturer’s instructions. Fluorescence was recorded as mean fluorescence intensity (MFI) and expressed relative to control conditions. For kinetic analyses, GSH-associated fluorescence was measured at multiple time points (0–24 h), and integrated responses were evaluated by calculating the area under the curve (AUC) from time-course MFI values. Cytoplasmic ROS levels were assessed using 2′,7′-dichlorodihydrofluorescein diacetate (DCF-DA; Sigma-Aldrich, Cat. No. D6883), mitochondrial mass using MitoTracker™ Green FM (Thermo Fisher Scientific, Cat. No. M7514), and mitochondrial superoxide production using MitoSOX™ Red (Thermo Fisher Scientific, Cat. No. M36008). For all probes, cells were incubated at 37 °C according to the manufacturers’ protocols, and fluorescence signals were acquired by flow cytometry. GSH, cytoplasmic ROS, and mitochondrial mass were expressed as mean fluorescence intensity (MFI) relative to control conditions, whereas mitochondrial superoxide levels were expressed as the percentage of MitoSOX-positive cells. Oxidative stress-related responses were assessed using complementary approaches, including intracellular glutathione (GSH) dynamics, total intracellular ROS, mitochondrial superoxide production, and the expression of antioxidant-related genes (GSR, GPX1, and CAT), in order to provide a broader evaluation of redox homeostasis under experimental conditions.

### 4.6. RNA Extraction and Quantitative Real-Time PCR (qPCR)

Total RNA was extracted using TRIzol™ reagent (Thermo Fisher Scientific, Cat. No. 15596026) according to the manufacturer’s protocol. RNA concentration and purity were assessed spectrophotometrically. Complementary DNA (cDNA) was synthesized from 1 μg of total RNA using a reverse transcription kit from Applied Biosystems (Thermo Fisher Scientific). Quantitative real-time PCR was performed using SYBR™ Green chemistry (Applied Biosystems, Thermo Fisher Scientific) on a QuantStudio™ Real-Time PCR System (Applied Biosystems, Thermo Fisher Scientific). Gene expression analyses included markers of EtOH metabolism (ADH, ALDH1, ALDH2), glutathione metabolism (GSR, GPX1), mitochondrial biogenesis (PGC-1α, TFAM), mitochondrial function and metabolic adaptation (SIRT1, PPARα, UCP2, COX-II), lipid metabolism (FAS), and inflammation (IL-6). Primer sequences used for qPCR amplification are reported in Supplementary Table S2. Relative gene expression was calculated using the 2^−ΔΔCt^ method and normalized to the housekeeping gene GAPDH.

### 4.7. Statistical Analysis

All experiments were performed in three independent biological replicates. For qPCR analyses, reactions were performed in technical triplicate and averaged before statistical analysis. For FACS analyses, all experiments were performed in two independent biological replicates and three different sample detections were carried out as technical replicates. Data are presented as mean ± standard deviation (SD). Statistical analyses were conducted using GraphPad Prism software 10. Data distribution normality and homogeneity of variance were assessed using Shapiro–Wilk and Levene tests, respectively. Differences among groups were evaluated using one-way analysis of variance (ANOVA) followed by Tukey’s multiple comparison post hoc test. Statistical significance was defined as * *p* < 0.05, ** *p* < 0.01, *** *p* < 0.001, and **** *p* < 0.0001.

## 5. Conclusions

In conclusion, PLE modulates hepatocellular responses to ethanol-induced stress through redox-sensitive and mitochondrial-related adaptive signatures rather than through direct antioxidant action. PLE was associated with improved oxidative handling and context-dependent modulation of mitochondrial and inflammatory pathways, while its effects on lipid metabolism and acetaldehyde-related responses require further investigation. Overall, these findings support the potential relevance of pistachio leaf-derived phytocomplexes as modulators of hepatocellular adaptive responses under redox stress conditions.

## Data Availability

The data presented in this study are available within the article and its Supplementary Materials.

## Supplementary Materials

The following supporting information can be downloaded at: https://www.mdpi.com/article/10.3390/ijms27114836/s1.

## Abbreviations

The following abbreviations are used in this manuscript:

EtOH	Ethanol
PLE	Pistachio Leaf Extract
ROS	Reactive Oxygen Species
GSH	Glutathione (reduced form)
SIRT1	Sirtuin 1
COX-II	Cytochrome c Oxidase Subunit II
FAS	Fatty Acid Synthase
IL-6	Interleukin-6
PGC-1α	Peroxisome proliferator-activated receptor gamma coactivator 1-alpha
PPARα	Peroxisome proliferator-activated receptor alpha
UCP2	Uncoupling Protein 2
ALDH1	Aldehyde Dehydrogenase 1
ALDH2	Aldehyde Dehydrogenase 2
ADH	Alcohol Dehydrogenase
GSR	Glutathione Reductase
GPX1	Glutathione Peroxidase 1
CAT	Catalase
TFAM	Mitochondrial Transcription Factor A
MFI	Mean Fluorescence Intensity
AUC	Area Under the Curve

## References

[1] Lieber C.S. (1991). Alcohol, Liver, and Nutrition. J. Am. Coll. Nutr..

[2] Massey V., Beier J., Ritzenthaler J., Roman J., Arteel G. (2015). Potential Role of the Gut/Liver/Lung Axis in Alcohol-Induced Tissue Pathology. Biomolecules.

[3] Kirpich I., Miller M., Cave M., Joshi-Barve S., McClain C. (2016). Alcoholic Liver Disease: Update on the Role of Dietary Fat. Biomolecules.

[4] Bailey S.M., Cunningham C.C. (2002). Contribution of Mitochondria to Oxidative Stress Associated with Alcoholic Liver Disease. Free Radic. Biol. Med..

[5] Mantena S.K., King A.L., Andringa K.K., Eccleston H.B., Bailey S.M. (2008). Mitochondrial Dysfunction and Oxidative Stress in the Pathogenesis of Alcohol- and Obesity-Induced Fatty Liver Diseases. Free Radic. Biol. Med..

[6] Kudo R., Adachi J., Uemura K., Maekawa T., Ueno Y., Yoshida K. (2001). Lipid Peroxidation in the Rat Brain after CO Inhalation Is Temperature Dependent. Free Radic. Biol. Med..

[7] Khan S.A., Sathyanarayan A., Mashek M.T., Ong K.T., Wollaston-Hayden E.E., Mashek D.G. (2015). ATGL-Catalyzed Lipolysis Regulates SIRT1 to Control PGC-1α/PPAR-α Signaling. Diabetes.

[8] Han D., Ybanez M.D., Johnson H.S., McDonald J.N., Mesropyan L., Sancheti H., Martin G., Martin A., Lim A.M., Dara L. (2012). Dynamic Adaptation of Liver Mitochondria to Chronic Alcohol Feeding in Mice. J. Biol. Chem..

[9] Manful C.F., Fordjour E., Ikumoinein E., Abbey L., Thomas R. (2025). Therapeutic Strategies Targeting Oxidative Stress and Inflammation: A Narrative Review. BioChem.

[10] Barbagallo I., Vanella L., Distefano A., Nicolosi D., Maravigna A., Lazzarino G., Di Rosa M., Tibullo D., Acquaviva R., Li Volti G. (2016). Moringa Oleifera Lam. Improves Lipid Metabolism during Adipogenic Differentiation of Human Stem Cells. Eur. Rev. Med. Pharmacol. Sci..

[11] Palmeri R., Monteleone J.I., Spagna G., Restuccia C., Raffaele M., Vanella L., Li Volti G., Barbagallo I. (2016). Olive Leaf Extract from Sicilian Cultivar Reduced Lipid Accumulation by Inducing Thermogenic Pathway during Adipogenesis. Front. Pharmacol..

[12] Rafiei H., Omidian K., Bandy B. (2019). Dietary Polyphenols Protect Against Oleic Acid-Induced Steatosis in an in Vitro Model of NAFLD by Modulating Lipid Metabolism and Improving Mitochondrial Function. Nutrients.

[13] Fuchs C., Bakuradze T., Steinke R., Grewal R., Eckert G.P., Richling E. (2020). Polyphenolic Composition of Extracts from Winery By-Products and Effects on Cellular Cytotoxicity and Mitochondrial Functions in HepG2 Cells. J. Funct. Foods.

[14] Gerdemann A., Broenhorst M., Behrens M., Humpf H.-U., Esselen M. (2023). Polyphenols Cause Structure Dependent Effects on the Metabolic Profile of Human Hepatocarcinogenic Cells. Mol. Nutr. Food Res..

[15] Wang T., Li M., Cai S., Zhou L., Hu X., Yi J. (2023). Polyphenol-Rich Extract of Fermented Chili Pepper Alleviates Insulin Resistance in HepG2 Cells via Regulating INSR, PTP1B, PPAR-γ, and AMPK Pathways. Fermentation.

[16] Wang L., Zou W., Zhong Y., An J., Zhang X., Wu M., Yu Z. (2012). The Hormesis Effect of BDE-47 in HepG2 Cells and the Potential Molecular Mechanism. Toxicol. Lett..

[17] Franco R., Navarro G., Martínez-Pinilla E. (2019). Hormetic and Mitochondria-Related Mechanisms of Antioxidant Action of Phytochemicals. Antioxidants.

[18] Benković V., Tkalčec I., Knežević A., Jurica K., Knežević F., Karačonji I.B., Kopjar N. (2024). Effects of Strawberry Tree (*Arbutus unedo* L.) Aqueous Leaf Extract and Arbutin on PK-15 and HepG2 Cells. Toxics.

[19] Mandalari G., Barreca D., Gervasi T., Roussell M.A., Klein B., Feeney M.J., Carughi A. (2021). Pistachio Nuts (*Pistacia vera* L.): Production, Nutrients, Bioactives and Novel Health Effects. Plants.

[20] Shahdadi F., Khorasani S., Salehi-Sardoei A., Fallahnajmabadi F., Fazeli-Nasab B., Sayyed R.Z. (2023). GC-MS Profiling of *Pistachio vera* L., and Effect of Antioxidant and Antimicrobial Compounds of It’s Essential Oil Compared to Chemical Counterparts. Sci. Rep..

[21] Marino G., Guzmán-Delgado P., Santos E., Adaskaveg J.A., Blanco-Ulate B., Ferguson L., Zwieniecki M.A., Fernández-Suela E. (2023). Interactive Effect of Branch Source-Sink Ratio and Leaf Aging on Photosynthesis in Pistachio. Front. Plant Sci..

[22] Farrokhi H., Eskandari M.H., Shakerardekani A., Golmakani M.T., Niakousari M. (2025). Pistachio Hull Extract as a Natural Antioxidant Incorporated into Pistachio Paste: Antioxidant Activity and Oxidative Stability. Appl. Food Res..

[23] Gentile C., Tesoriere L., Butera D., Fazzari M., Monastero M., Allegra M., Livrea M.A. (2007). Antioxidant Activity of Sicilian Pistachio (*Pistacia vera* L. Var. Bronte) Nut Extract and Its Bioactive Components. J. Agric. Food Chem..

[24] Tomaino A., Martorana M., Arcoraci T., Monteleone D., Giovinazzo C., Saija A. (2010). Antioxidant Activity and Phenolic Profile of Pistachio (*Pistacia vera* L., Variety Bronte) Seeds and Skins. Biochimie.

[25] Massimo L., Laura D., Ginevra L.-B. (2020). Phytosterols and Phytosterol Oxides in Bronte’s Pistachio (*Pistacia vera* L.) and in Processed Pistachio Products. Eur. Food Res. Technol..

[26] Sferrazzo G., Palmeri R., Restuccia C., Parafati L., Siracusa L., Spampinato M., Carota G., Distefano A., Di Rosa M., Tomasello B. (2022). *Mangifera indica* L. Leaves as a Potential Food Source of Phenolic Compounds with Biological Activity. Antioxidants.

[27] Babaeian M., Tavassoli A., Rastegaripour F., Rodrigo-Comino J., Caballero-Calvo A. (2025). Analysis of Energy Use and Environmental Impacts of Pistachio (*Pistacia vera* L.) in Conventional and Bio-Friendly Production Systems. Appl. Fruit Sci..

[28] Koul B., Yakoob M., Shah M.P. (2022). Agricultural Waste Management Strategies for Environmental Sustainability. Environ. Res..

[29] Fermanelli C.S., Chiappori A., Pierella L.B., Saux C. (2022). Towards Biowastes Valorization: Peanut Shell as Resource for Quality Chemicals and Activated Biochar Production. Sustain. Environ. Res..

[30] Bhatia L., Kaladhar D.S.V.G.K., Sarkar T., Jha H., Kumar B. (2024). Food Wastes Phenolic Compounds (PCs): Overview of Contemporary Greener Extraction Technologies, Industrial Potential, and Its Integration into Circular Bioeconomy. Energy Ecol. Environ..

[31] Batovska D., Inbar M. (2024). Beyond the Nut: Pistacia Leaves as Natural Food Preservatives. Foods.

[32] Elakremi M., Sillero L., Ayed L., ben Mosbah M., Labidi J., ben Salem R., Moussaoui Y. (2022). *Pistacia vera* L. Leaves as a Renewable Source of Bioactive Compounds via Microwave Assisted Extraction. Sustain. Chem. Pharm..

[33] Spampinato M., Furnari S., Siracusa L., Malfa G.A., Distefano A., La Spina E., Gulisano M., Furneri P.M., Fuochi V., Barbagallo I.A. (2025). Pistachio leaf waste transformed into a gut-targeted bioactive phytocomplex. iScience.

[34] Hokmabadi H., Arzani K., Grierson P.F. (2005). Growth, Chemical Composition, and Carbon Isotope Discrimination of Pistachio (*Pistacia vera* L.) Rootstock Seedlings in Response to Salinity. Aust. J. Agric. Res..

[35] Moreno-Rojas J.M., Velasco-Ruiz I., Lovera M., Ordoñez-Díaz J.L., Ortiz-Somovilla V., De Santiago E., Arquero O., Pereira-Caro G. (2022). Evaluation of Phenolic Profile and Antioxidant Activity of Eleven Pistachio Cultivars (*Pistacia vera* L.) Cultivated in Andalusia. Antioxidants.

[36] Gok H.N., Pekacar S., Deliorman Orhan D. (2022). Investigation of Enzyme Inhibitory Activities, Antioxidant Activities, and Chemical Properties of *Pistacia vera* Leaves Using LC-QTOF-MS and RP-HPLC. Iran. J. Pharm. Res..

[37] Koch O.R., Fusco S., Ranieri S.C., Maulucci G., Palozza P., Larocca L.M., Cravero A.A.M., Farre’ S.M., De Spirito M., Galeotti T. (2008). Role of the Life Span Determinant P66shcA in Ethanol-Induced Liver Damage. Lab. Investig..

[38] Lamlé J., Marhenke S., Borlak J., Von Wasielewski R., Eriksson C.J.P., Geffers R., Manns M.P., Yamamoto M., Vogel A. (2008). Nuclear Factor-Eythroid 2–Related Factor 2 Prevents Alcohol-Induced Fulminant Liver Injury. Gastroenterology.

[39] Larosche I., Lettéron P., Berson A., Fromenty B., Huang T.-T., Moreau R., Pessayre D., Mansouri A. (2010). Hepatic Mitochondrial DNA Depletion after an Alcohol Binge in Mice: Probable Role of Peroxynitrite and Modulation by Manganese Superoxide Dismutase. J. Pharmacol. Exp. Ther..

[40] Guru A., Manjunathan T., Sudhakaran G., Juliet A., Gopinath P., Arockiaraj J. (2023). 6-Gingerdione Reduces Apoptotic Conditions in HepG2 Cells and Inhibits Inflammatory Cytokine Gene Expression in Alcoholic Liver Injured Zebrafish Larvae. Chem. Biodivers..

[41] Barbagallo I., Tibullo D., Di Rosa M., Giallongo C., Palumbo G.A., Raciti G., Campisi A., Vanella A., Green C.J., Motterlini R. (2008). A Cytoprotective Role for the Heme Oxygenase-1/CO Pathway during Neural Differentiation of Human Mesenchymal Stem Cells. J. Neurosci. Res..

[42] Barbagallo I., Marrazzo G., Frigiola A., Zappala A., Li Volti G. (2012). Role of Carbon Monoxide in Vascular Diseases. Curr. Pharm. Biotechnol..

[43] Ristow M., Schmeisser K. (2014). Mitohormesis: Promoting Health and Lifespan by Increased Levels of Reactive Oxygen Species (ROS). Dose Response.

[44] Almatroodi S.A., Rahmani A.H. (2025). Unlocking the Pharmacological Potential of Myricetin Against Various Pathogenesis. Int. J. Mol. Sci..

[45] Akter R., Kwak G.-Y., Ahn J.C., Mathiyalagan R., Ramadhania Z.M., Yang D.C., Kang S.C. (2021). Protective Effect and Potential Antioxidant Role of Kakadu Plum Extracts on Alcohol-Induced Oxidative Damage in HepG2 Cells. Appl. Sci..

[46] Song J., Kim Y., Lee J. (2018). Comparison of Antioxidant and Anti-Inflammatory Activity of Quercetin, Isoquercitrin and Rutin against Alcohol-Induced Liver Injury in HepG2 Cells. FASEB J..

[47] Lee Y.-J., Beak S.-Y., Choi I., Sung J.-S. (2017). Quercetin and Its Metabolites Protect Hepatocytes against Ethanol-Induced Oxidative Stress by Activation of Nrf2 and AP-1. Food Sci. Biotechnol..

[48] Puttahanumantharayappa L.D., Sannappagowda N.G., Shiragannanavar V.D., Karunakara S.H., Santhekadur P.K. (2022). Ameliorating Effect of Quercetin on Ethanol-Induced Liver Injury Via Targeting RISC Machinery. Int. J. Health Allied Sci..

[49] Zhou Q., Wang L., Liu B., Xiao J., Cheng K.-W., Chen F., Wang M. (2021). Tricoumaroylspermidine from Rose Exhibits Inhibitory Activity against Ethanol-Induced Apoptosis in HepG2 Cells. Food Funct..

[50] Silva J., Spatz M.H., Folk C., Chang A., Cadenas E., Liang J., Davies D.L. (2021). Dihydromyricetin Improves Mitochondrial Outcomes in the Liver of Alcohol-Fed Mice via the AMPK/Sirt-1/PGC-1α Signaling Axis. Alcohol..

[51] Longhitano L., Distefano A., Musso N., Bonacci P., Orlando L., Giallongo S., Tibullo D., Denaro S., Lazzarino G., Ferrigno J. (2024). (+)-Lipoic Acid Reduces Mitochondrial Unfolded Protein Response and Attenuates Oxidative Stress and Aging in an in Vitro Model of Non-Alcoholic Fatty Liver Disease. J. Transl. Med..

[52] Naghdi S., Slovinsky W.S., Madesh M., Rubin E., Hajnóczky G. (2018). Mitochondrial Fusion and Bid-Mediated Mitochondrial Apoptosis Are Perturbed by Alcohol with Distinct Dependence on Its Metabolism. Cell Death Dis..

[53] Srinivasan M., Kota S., Bhopale K., Caracheo A., Kaphalia L., Linares J., Romsdahl T., Russell W., Popov V., Boor P. (2025). Dysregulated Hepatic Alcohol Metabolism: A Key Factor Involved in the Pathogenesis of Alcohol-Associated Liver Disease. Am. J. Physiol.-Gastrointest. Liver Physiol..

[54] Li J., Shi X., Chen Z., Xu J., Zhao R., Liu Y., Wen Y., Chen L. (2023). Aldehyde Dehydrogenase 2 Alleviates Mitochondrial Dysfunction by Promoting PGC-1α-Mediated Biogenesis in Acute Kidney Injury. Cell Death Dis..

[55] Longhitano L., Li Volti G., Giallongo C., Spampinato M., Barbagallo I., Di Rosa M., Romano A., Avola R., Tibullo D., Palumbo G.A. (2020). The Role of Inflammation and Inflammasome in Myeloproliferative Disease. J. Clin. Med..

