# Supplementary material for: Pistachio Leaf Extract Modulates Redox-Dependent Mitochondrial and Metabolic Responses in Ethanol-Stressed HepG2 Cells
Source: Int J Mol Sci. 2026 May 27;27(11):4836. doi: 10.3390/ijms27114836 (PMC13256378; doi:10.3390/ijms27114836)
Supplement: Supplementary file 1 [file ijms-27-04836-s001.zip › ijms-4314105-supplementary.pdf]

Supplementary Materials:

Supplementary Table S1. **Quantitative chemical characterization of** PLE. Main specialized metabolites identified in PLE, and their quantitative content expressed as mg/100 mg dry extract. S1 is adapted from our previously published work [33], which reported the full chemical characterisation of the extract. The present version summarises the major compounds relevant to the biological assays.

**Peak**	**RT (min)**	**Tentative identification**	**Molecular formula**	**Exact mass**	**Exact mass**	**mg/100**
**1**	4.639	Gallic acid	$C_7H_6O_5$	170.0215	169.08	2.681
**2**	17.966	Quercetin di-hexoside	$C_{27}H_{30}O_{17}$	626.1483	625.51	0.059
**3**	18.646	Quercetin galloyl-hexoside 1	$C_{28}H_{24}O_{16}$	616.1064	615.00	0.070
**4**	18.944	Quercetin hexoside derivative	–	–	493.29	0.611
**5**	19.443	Quercetin galloyl-hexoside 2	$C_{28}H_{24}O_{16}$	616.1064	615.07	0.067
**6**	19.690	Quercetin galloyl-hexoside 3	$C_{28}H_{24}O_{16}$	616.1064	615.84	0.062
**7**	20.028	Quercetin hexoside–deoxyhexosic	$C_{27}H_{30}O_{17}$	610.1533	609.92	0.196
**8**	20.690	Quercetin glucuronide	$C_{21}H_{18}O_{13}$	478.0747	477.07	0.390
**9**	20.965	Myricetin hexoside 1	$C_{21}H_{20}O_{13}$	480.0903	479.00	0.038
**10**	21.193	Myricetin hexoside 2	$C_{21}H_{20}O_{13}$	480.0903	479.00	0.032
**11**	21.257	Anacardic acid (C17:1)	$C_{24}H_{38}O_3$	374.2820	373.50	0.144
**12**	21.755	Quercetin 3-O-glucoside	$C_{21}H_{20}O_{12}$	464.0954	463.58	0.098
**13**	21.887	Myricetin deoxyhexoside	$C_{21}H_{20}O_{12}$	464.0954	463.17	0.135
**14**	22.846	Myricetin galloyl glucuronide	$C_{28}H_{22}O_{18}$	646.0806	645.09	0.090
**15**	23.067	Myricetin galloyl hexoside	$C_{28}H_{24}O_{17}$	632.1013	631.58	0.028
**16**	23.481	Myricetin galloyl deoxyhexoside	$C_{28}H_{24}O_{16}$	616.1064	615.25	0.012
**17**	24.510	Myricetin digalloyl deoxyhexosid	$C_{35}H_{28}O_{20}$	768.1174	767.17	0.011

Supplementary Table S2. Primer sequences used for quantitative real-time PCR

Gene	Forward primer (5′–3′)	Length (nt)	Reverse primer (5′–3′)	Length (nt)
GAPDH	CCTGCACCACCAACTGCTA	20	GGCCATCCACAGTCTTCTGAG	21
ADH1	CTCGCCCCTGGAGAAAGTC	19	GGCCCCCAACTCTTTAGCC	19
ALDH1A1	GCACGCCAGACTTACCTGTC	20	CCTCCTCAGTTGCAGGATTAAAG	23
ALDH2	ATGGCAAGCCCTATGTCATCT	21	CCGTGGTACTTATCAGCCCA	20
GSR	AGGCTTCCTGCTGCTTCTG	19	CAACATTCACGCAAGTGCCA	20
GPX1	CAGTCGGTGTATGCCTTCTCG	21	GAGGGACGCCACATTCTCG	19

PGC-1α	TCTGAGTCTGTATGGAGTGACAT	23	CCAAGTCGTTCACATCTAGTTCA	22
TFAM	CCGAGGTGGTTTTCATCTGT	20	AGTCTTCAGCTTTTCCTGCG	20
SIRT1	GCCTCACATGCAAGCTCTAGTGAC	24	TTCGAGGATCTGTGCCAATCATA A	24
PPARα	CATACTCGCGGGAAAGACCA	20	CGTCTTCTCGGCCATACACA	20
COX-II	TGCCCTTTTCCTAACACTCACAA	23	CGCCGTAGTCGGTGTACTCG	20
FAS	ACAGGTGGGAACAAGG	16	ATGAAGAGGGACCATAAA	18
IL-6	GAAAGCAGCAAAGAGGCACT	20	TTTCACCAGGCAAGTCTCCT	20
CAT	GTGCGGAGATTCAACACTGCCA	22	CGGCAATGTTCTCACACAGACG	22
